# Towards sustainable healthcare in otorhinolaryngology: assessing knowledge, engagement, and opportunities for change

**DOI:** 10.3389/frhs.2026.1867484

**Published:** 2026-07-17

**Authors:** Ilona Anderson, Aanand Acharya, Uwe Baumann, Kevin D. Brown, Miryam Calvino, Marco Caversaccio, Javier Gavilán, Sara Ghiselli, Benoit Godey, Rudolf Hagen, Abdulrahman Hagr, Emilie Heuninck, Mohan Kameswaran, Anja Kurz, Marc Lammers, Yongxin Li, Griet Mertens, Robert Mlynski, Martin O'Driscoll, Sasidharan Pulibalathingal, Christopher H. Raine, Ranjith Rajeswaran, Kristen Rak, Joachim Schmutzhard, Josef Seebacher, Piotr H. Skarzynski, Kari Smilsky, Georg M. Sprinzl, Hinrich Staecker, Timo Stöver, David Strachan, Serafima Sugarova, Paul Van de Heyning, Vincent Van Rompaey, Christiane Völter, Medhat Youssef, Mario E. Zernotti, Kim Zimmerman, Christiana Kyvelidou

**Affiliations:** 1 Clinical Research Department, MED-EL GmbH, Innsbruck, Austria; 2 Department of Otolaryngology Head and Neck Surgery, Fiona Stanley Fremantle Hospital Group, Perth, WA, Australia; 3 ENT/Audiological Acoustics, Goethe University Frankfurt, University Hospital, Frankfurt am Main, Germany; 4 Department of Otolaryngology-Head and Neck Surgery, UNC Ear & Hearing Center at Chapel Hill School of Medicine, Chapel Hill, NC, United States; 5 Department of Otorhinolaryngology, Hospital La Paz, IdiPAZ, Madrid, Spain; 6 Biomedical Research Networking Centre on Rare Diseases (CIBERER), Institute of Health Carlos III, (CIBERER-U761), Madrid, Spain; 7 Department for ENT Head and Neck Surgery, Bern University Hospital, Bern, Switzerland; 8 Department of Otolaryngology, AUSL Piacenza, Piacenza, Italy; 9 Department of Medicine and Surgery, University of Parma, Parma, Italy; 10 Department of Otolaryngology–Head and Neck Surgery, University Hospital of Rennes, Rennes, France; 11 Department of Oto-Rhino-Laryngology Head and Neck Surgery, University of Würzburg, Würzburg, Germany; 12 King Abdullah Ear Specialist Center (KAESC), College of Medicine, King Saud University, Riyadh, Saudi Arabia; 13 Department of Otorhinolaryngology Head and Neck Surgery, University Hospital UZ Brussel, Vrije Universiteit Brussel, Brussels, Belgium; 14 Audiology Department, Madras ENT Research Foundation (MERF), Chennai, Tamil Nadu, India; 15 Department of Otolaryngology, University Hospital Würzburg, Comprehensive Hearing Center, Würzburg, Germany; 16 Department of Otorhinolaryngology Head and Neck Surgery, Antwerp University Hospital (UZA), Antwerp, Belgium; 17 Department of Translational Neurosciences Resonant Labs Antwerp Faculty of Medicine and Health Sciences, University of Antwerp, Antwerp, Belgium; 18 Department of Otology, Beijing Tongren Hospital, Capital Medical University, Beijing, China; 19 Department of Otorhinolaryngology Head and Neck Surgery, ‘Otto-Koerner’, University of Rostock, Rostock, Germany; 20 Manchester Auditory Implant, Central Manchester University Hospitals, Manchester, United Kingdom; 21 Dr Manoj’s ENT Super Speciality Institute and Research Centre Center, Kozhikode, Kerala, India; 22 Bradford Royal Infirmary, Yorkshire Auditory Implant Service, Bradford, United Kingdom; 23 Department of Otorhinolaryngology Head and Neck Surgery, Medical University of Innsbruck, Innsbruck, Austria; 24 Department for Hearing Speech & Voice Disorders, Medical University of Innsbruck, Innsbruck, Austria; 25 Department of Teleaudiology and Screening, World Hearing Center, Institute of Physiology and Pathology of Hearing, Warsaw, Poland; 26 Institute of Sensory Organs, Kajetany, Poland; 27 Sunnybrook Cochlear Implant Program, Sunnybrook Health Sciences Centre, Toronto, ON, Canada; 28 Department of Otorhinolaryngology Head & Neck Surgery, St. Pölten University Hospital, St. Pölten, Austria; 29 ENT Department, University of Kansas Medical Centre, Kansas, MO, United States; 30 Department of Otolaryngology, Goethe University Frankfurt, University Hospital, Frankfurt am Main, Germany; 31 St. Petersburg Research Institute of Ear Nose Throat and Speech, St Petersburg, Russia; 32 Department of Otorhinolaryngology Head and Neck Surgery, St. Elisabeth Hospital, Ruhr University, Bochum, Germany; 33 Department of Otorhinolaryngology, Sanatorio Allende de Córdoba, Córdoba, Argentina; 34 Catholic University of Córdoba and National University of Córdoba, Córdoba, Argentina; 35 Cochlear Implant Program, London Health Sciences Centre, London, ON, Canada

**Keywords:** cochlear implants, green teams, healthcare sustainability, hearing care, otorhinolaryngology (ORL)

## Abstract

**Introduction:**

Healthcare systems contribute up to 5% of global greenhouse gas emissions, with impacts varying across medical specialties. Otorhinolaryngology (ORL) and hearing care must address their specific environmental footprint to meet emerging sustainability goals. This study assessed ORL professionals’ awareness, engagement, and perceived opportunities for implementing sustainable practices.

**Methods:**

A 17-item survey was conducted from May 31 to July 25, 2024, among members of the HEARRING group, an international association of ORL experts.

**Results:**

37 responses from 26 hospitals across 16 countries were collected. While 89.2% of participants expressed interest in sustainability, only 29.7% felt confident explaining sustainable development. Key opportunities included waste reduction (70%), policy development (25%), transportation improvements (20%), and energy efficiency (15%). Major barriers were lack of collective action (59.5%), uncertainty (35.1%), and insufficient workplace support (35.1%). Hospitals commonly engaged in recycling (83.3%), waste reduction (80.6%), and energy conservation (74.3%), but green purchasing and toxic-waste reduction were limited. Awareness of Green Teams was low (30.6%), though those familiar recognized their value.

**Discussion:**

Despite strong interest, gaps in knowledge and systemic barriers persist in ORL care. Targeted education, leadership support, and adoption of Green Teams could accelerate sustainable practices and reduce environmental impact.

## Graphical Abstract

**Figure:**
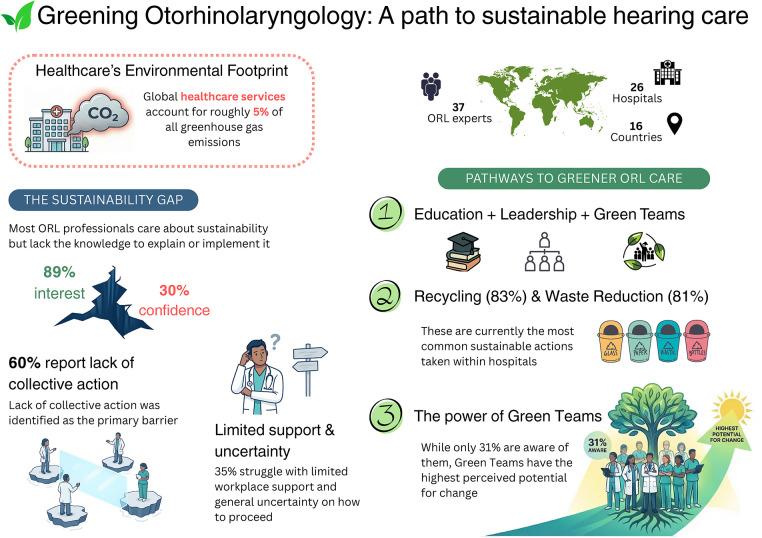

## 1 Introduction

The environmental impact of healthcare is a matter of increasing global concern (1). Approximately 4%–5% of global greenhouse emissions are attributed to healthcare systems. The majority of these represent indirect emissions, predominantly connected to the use of disposable materials, medical and non-medical equipment, and pharmaceuticals (2). Promoting sustainability within healthcare is essential for mitigating climate change and its associated health risks (3). Aligning healthcare practices with the United Nations' Sustainable Development Goals (SDGs), particularly Goal 3 (Good Health and Well-Being) and Goal 13 (Climate Action), is a vital step toward addressing the sector's environmental footprint (4).

To better quantify and understand these environmental impacts, Life Cycle Assessment (LCA) has been developed as a key methodological framework. LCA is a standardised approach for evaluating the environmental impacts associated with all the stages of a product, process, or service which can be applied to healthcare systems to support evidence-based sustainability decisions (5, 6). In hospitals, LCA enables comparison between alternative practices, such as reusable vs. single-use devices, and helps identify areas for improvement (7).

Medical practices, particularly in high-resource settings like operating theatres, often generate large amounts of waste, consume substantial resources, and contribute to pollution (8). However, the nature and magnitude of environmental impact vary across medical specialties due to differences in resource usage, waste generation, and energy demands. Recognizing these variations, recent studies have called for specialty-specific strategies to tackle sustainability challenges effectively (9–12).

An audit of invasive cardiac procedures reported an annual production of approximately 11,000 kg of recyclable waste and 30,000 kg of contaminated waste in a single centre, urging cardiologists to reduce waste and prioritize recycling efforts to mitigate the sector's environmental burden (9). In surgical pathology, sustainable solutions—such as the integration of digital technologies, circular economy principles, and green innovations—are being explored (10). Intensive care units have demonstrated the effectiveness of targeted educational interventions; for instance, reducing the unnecessary usage of intermittent-pneumatic-compression devices resulted in a reduction of 51.8 kg CO_2_ equivalent (CO_2_e) annually, alongside decreases in waste production and costs, proving the power of behaviour-change interventions. Orthopaedic surgery provides further examples of initiatives to mitigate environmental impact, such as reusing materials—like external fixators—and implementing closed-loop supply chains (11). Nevertheless, ethical concerns, such as the necessity for informed consent when reusing medical devices, call for greater transparency (11). In dialysis centres, where carbon emissions from haemodialysis are estimated at 12.8 tons CO_2_e per person annually, frameworks targeting prevention, green technologies, and improved access to transplantation are proposed (12).

Education is critical for fostering sustainable practices. Integrating sustainability principles into radiography curricula has proven effective in fostering a culture of environmental responsibility among healthcare professionals, equipping them to implement ecologically sound practices and advocate for global health (13). Similar educational initiatives have been developed in postgraduate medical training and surgical fellowship programs (14, 15). Collectively, these examples across cardiology, pathology, intensive care, orthopaedics, dialysis care, and radiography affirm the need for each medical specialty to identify its unique environmental challenges and to develop targeted and pragmatic solutions.

Otorhinolaryngology (ORL) encompasses high-volume outpatient activity and surgical care, including endoscopic examinations and operating theatre procedures that, as in many other specialties, commonly rely on disposable consumables, instrument processing, and energy-intensive clinical environments (16, 17). Hearing care and cochlear implantation additionally involve device-related supply chains, packaging, and ongoing follow-up care that may require repeated patient travel (18). These features make ORL well positioned for targeted sustainability interventions across clinical pathways, procurement, and service delivery.

In this respect, hearing care and ORL must address their environmental impact by identifying areas for improvement and integrating sustainable practices into routine clinical care. The primary aim of this survey was to assess ORL professionals’ current understanding of and engagement with sustainability efforts. Furthermore, it sought to find opportunities for implementing sustainable practices within the field.

## 2 Materials and methods

### 2.1 Participants

Participants for the survey were drawn from the HEARRING group, an international association of ORL experts with a focus on cochlear implants (19). All members of the HEARRING group were invited to participate in the survey via email invitation and were not pre-selected based on any criteria. A total of 77 invitations were sent.

Prior to analysis, survey responses were extracted and anonymised, and all direct identifiers were removed from the analytical dataset. As a result, individual responses could not be linked to specific participants during analysis, and survey findings are presented only in aggregated form. Survey respondents were subsequently invited to be co-authors if they fulfilled the International Committee of Medical Journal Editors (ICMJE) authorship criteria. While participant identities were known within the collaborative network, individual survey responses were not identifiable within the analysed dataset or reported results.

### 2.2 Ethics statement

This was a voluntary, non-interventional survey of healthcare professionals regarding their attitudes and practices towards sustainability. No patient data or medical records were accessed. There was no credible risk of physical, psychological, or informational harm to the participants. Ethical review and approval was not required for the study of human participants in accordance with the local legislation, institutional requirements and the characteristics of the study (Austrian Medicines Act, § 29 AMG; the Federal Hospitals Act, § 8c KAKuG; and the Universities Act, § 30 UG 2002). The voluntary completion and return of the survey constituted implied consent via conclusive action under Austrian Civil Law (ABGB) Section 863 (1). Thus, written informed consent from participants was not required to participate in this study in accordance with the national legislation, the institutional requirements, and the characteristics of the study.

### 2.3 Survey details

The survey was implemented using the Qualtrics XMcloud-based software/web platform. Data were collected between May 31, 2024, and July 25, 2024. The survey comprised a total of 17 items in total, organized into several sections and was conducted in English. Participants were not required to answer every survey item; therefore, response totals vary slightly across questions.

#### 2.3.1 Basic demographic and sustainability habits

Participants were asked to provide basic personal information including their name, the city where their main clinical practice is, and their age range. Participants were then queried on their interest and understanding of sustainability. Participants were asked whether they a. are interested in sustainability, b. are unsure what sustainable development means, and c. feel confident explaining sustainable development as a concept. Possible answers were *yes*, *no*, or *partially*. Participants were asked if they saw opportunities within their current role to help their hospital become a more sustainable organization, and if they did, they were asked to list the opportunities they saw. Participants were asked if they had experience in working on sustainability related projects and/or initiatives, and if they had, they were asked to describe them. Participants were asked to reflect on their sustainability-related actions at work. The response options are shown in Additional files (Additional Supplementary Table S1: Workspace actions that relate to sustainability). Possible answers were *yes*, *sometimes*, or *no*. Participants were asked to identify barriers to adopting sustainable practices at work; a list of possible barriers from which participants could choose is shown in Additional files (Additional Supplementary Table S2: Barriers to considering a more sustainable environment at work). Participants could also select *Other* to provide additional items that they considered as barriers.

#### 2.3.2 Hospital sustainability actions

In this section, participants’ awareness and engagement with their hospital's sustainable practices were explored. Participants were asked if they were aware of any sustainable practices currently implemented in their hospital. Participants were asked to rate any sustainability activities taken by their hospital according to a five-point Likert scale. Possible answers were *0 – don't know*, *1 – none*, *2 – a little*, *3 – quite a bit*, and *4 – a great deal*. A list with the sustainability activities is shown in Additional files (Additional Supplementary Table S3: Barriers to considering a more sustainable environment at work). Participants could also select *Other* to provide additional sustainability actions. Participants were asked if they believe the hospital should invest more in sustainable healthcare technologies, if there are available educational programs about sustainability for hospital staff and patients, and if they would participate in initiatives or programs promoting sustainability within the hospital.

#### 2.3.3 Green team awareness

The participants’ familiarity with the concept of a ‘Green Team’ was assessed. A Green Team is a group of employees within an organization—often from diverse departments and levels—who collaborate to promote and implement environmentally sustainable practices in the workplace (20). These teams typically focus on reducing waste, conserving energy, encouraging sustainable procurement, and fostering a culture of environmental responsibility. Participants were asked if they had ever heard of a Green Team, and if yes, who should be in a Green Team. Participants could choose one of the following: *the CEO*, *the dean*, *middle management*, and *employees*. Then participants were asked to explain what they think a Green Team does. Finally, participants were given the opportunity to provide suggestions on enhancing workplace sustainability.

### 2.4 Statistical methods

Descriptive statistics were used to report participants’ characteristics and survey results. Responses are presented as absolute numbers and percentages. For open-ended questions, responses were categorized based on content and summarized accordingly. Missing data were treated as missing values. Statistical analyses were conducted in Microsoft Excel (Microsoft 365, version 2402; Microsoft Corporation, Redmond, WA).

## 3 Results

### 3.1 General information and demographics

A total of 37 surveys were collected (response rate 48.1%; fully completed 97.3%) from ORL professionals. Of the respondents, 35.1% (*n* = 13) were audiologists/clinical engineers, and 64.9% (*n* = 24) were surgeons. Participants were drawn from 26 hospitals across 16 countries, spanning 4 continents (Oceania, Europe, North America, and Asia). Most participants (63.9%; 23 out of 36) were in the 40–59 years age range.

### 3.2 Insights on sustainability awareness, opportunities, and engagement

**Figure 1:**
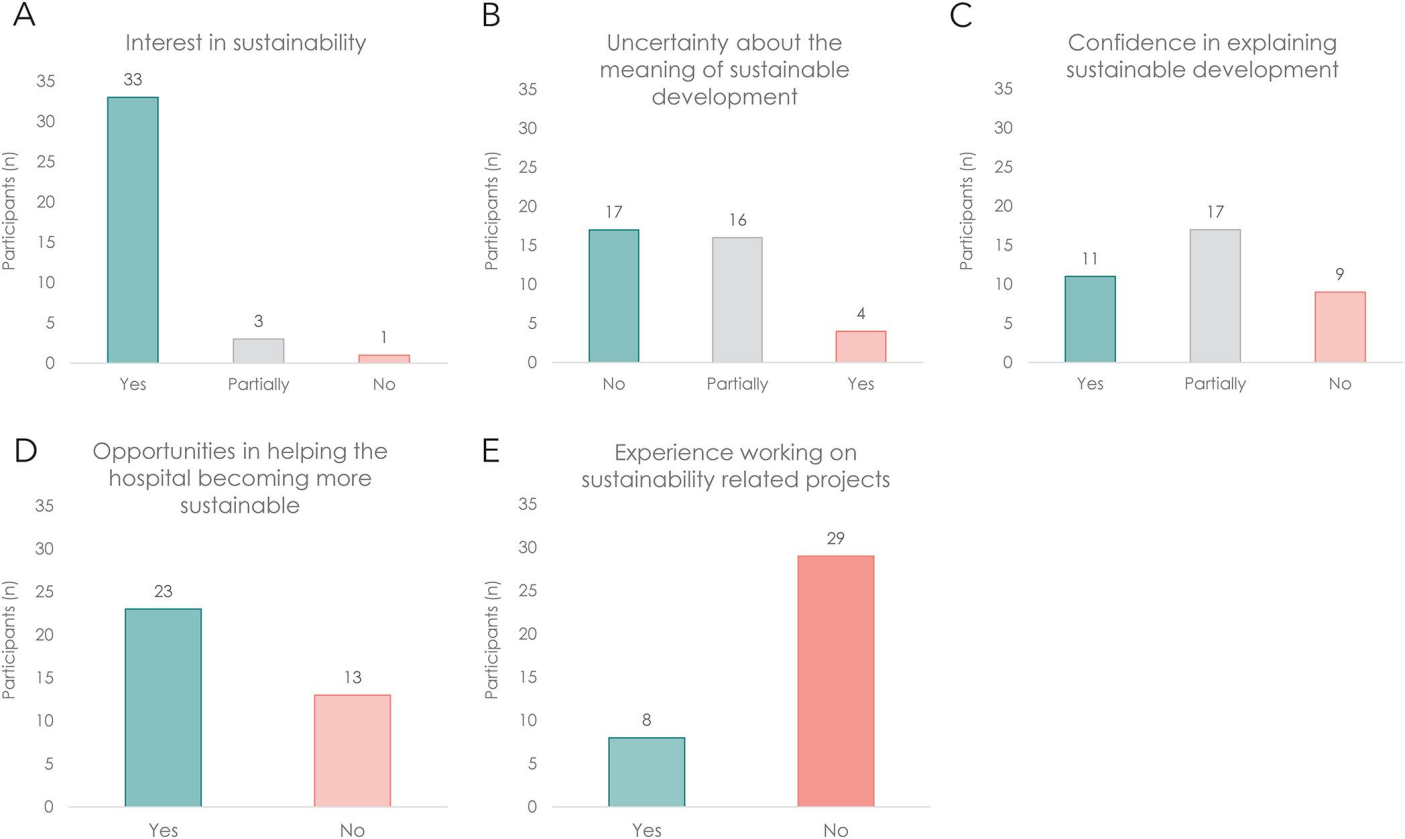
Participants’ answers regarding sustainability awareness, opportunities, and engagement. **(A)** Are you interested in sustainability? (*n* = 37); **(B)** Are you unsure about what sustainable development means? (*n* = 37); **(C)** Do you feel confident explaining sustainable development as a concept? (*n* = 37); **(D)** Do you see opportunities within your current role to help your hospital become a more sustainable organization? (*n* = 36); **(E)** Do you have experience working on sustainability related projects/initiatives? (*n* = 37), *y*-axis represents the absolute number of participants.

Most participants (89.2%; 33 out of 37) were interested in sustainability (Figure 1A), and nearly half (45.9%; 17 out of 37) were clear on what sustainable development means (Figure 1B). However, less than a third (29.7%; 11 out of 37) felt confident explaining the concept of sustainable development (Figure 1C).

Two thirds of participants (63.9%; 23 out of 36) recognized opportunities within their current roles to contribute to their hospital's sustainability efforts (Figure 1D). When asked to describe these opportunities, waste reduction and recycling were the most consistently mentioned (70%; 14 out of 20), followed by developing new policies and advocating sustainability (25%; 5 out of 20), transportation (20%; 4 out of 20), and energy efficiency (15%; 3 out of 20). Waste reduction opportunities included recycling hearing aid devices, reducing printing and promoting a paperless office, minimizing packaging, using more reusable materials instead of single-use items—such as gowns, drapes, and burs—and improving waste separation. On a personal level, some participants recognized opportunities, such as reusing paper boxes for the mail and encouraging team members to avoid disposable cups. Advocacy opportunities included engaging in interprofessional discussions, addressing issues with the medical director, or actively developing policies to enable sustainability in the hospital. Transportation opportunities involved reducing travel for patients by promoting remote clinic appointments and encouraging remote work and meetings for healthcare professionals. Finally, energy efficiency opportunities included adopting sustainable power solutions and promoting energy conservation.

One fifth of the participants (21.6%; 8 out of 37) had experience working on sustainability-related projects or initiatives (Figure 1E). These projects involved establishing a green operating theatre, participating in and promoting sustainable projects such as the ‘Green Cochlea’ (21), following specific guidelines, or reducing the hospital's carbon footprint through the installation of solar panels.

**Figure 2:**
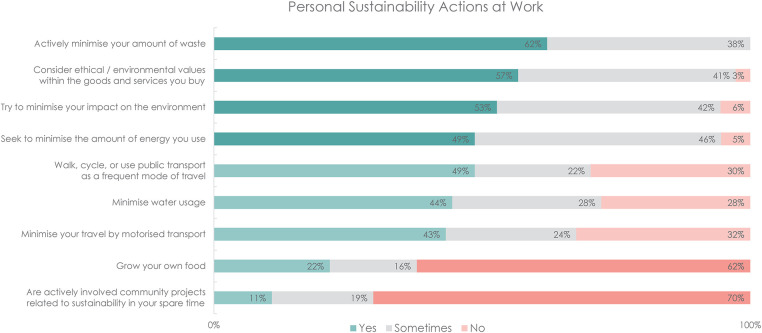
Workspace actions that relate to sustainability. As a reflection of their actions at work, participants were asked to indicate where they consistently performed these actions, performed them sometimes, or consistently did not perform them; (*n* = 37 except for ‘Try to minimize your impact on the environment’ and ‘Minimize water usage’ where *n* = 36), *x*-axis represents the percentage of participants.

Survey participants were asked to reflect on their sustainability-related workspace actions and indicate whether they consistently performed these actions, performed them sometimes, or consistently did not perform them (Figure 2). Participants showed strong engagement (more than 90% either consistently or sometimes performing these actions) in waste reduction, considering ethical or environmental values when purchasing goods and services, minimizing their environmental impact, and minimizing their energy use. Nearly half of the participants indicated that they always try to walk, cycle, or use public transportation as a frequent mode of travel (48.6%; 18 out of 37), and two out of five always try to minimize travel by motorized transport (43.2%; 16 out of 37) and reduce water usage (44.4%; 16 out of 36). In contrast, the lowest levels of active engagement were seen in activities such as growing one's own food (78.4%; 23 out of 37) and participating in community sustainability projects (89.2%; 26 out of 37), with more than three-quarters of participants not consistently performing these actions.

### 3.3 Insights on sustainability barriers

**Figure 3:**
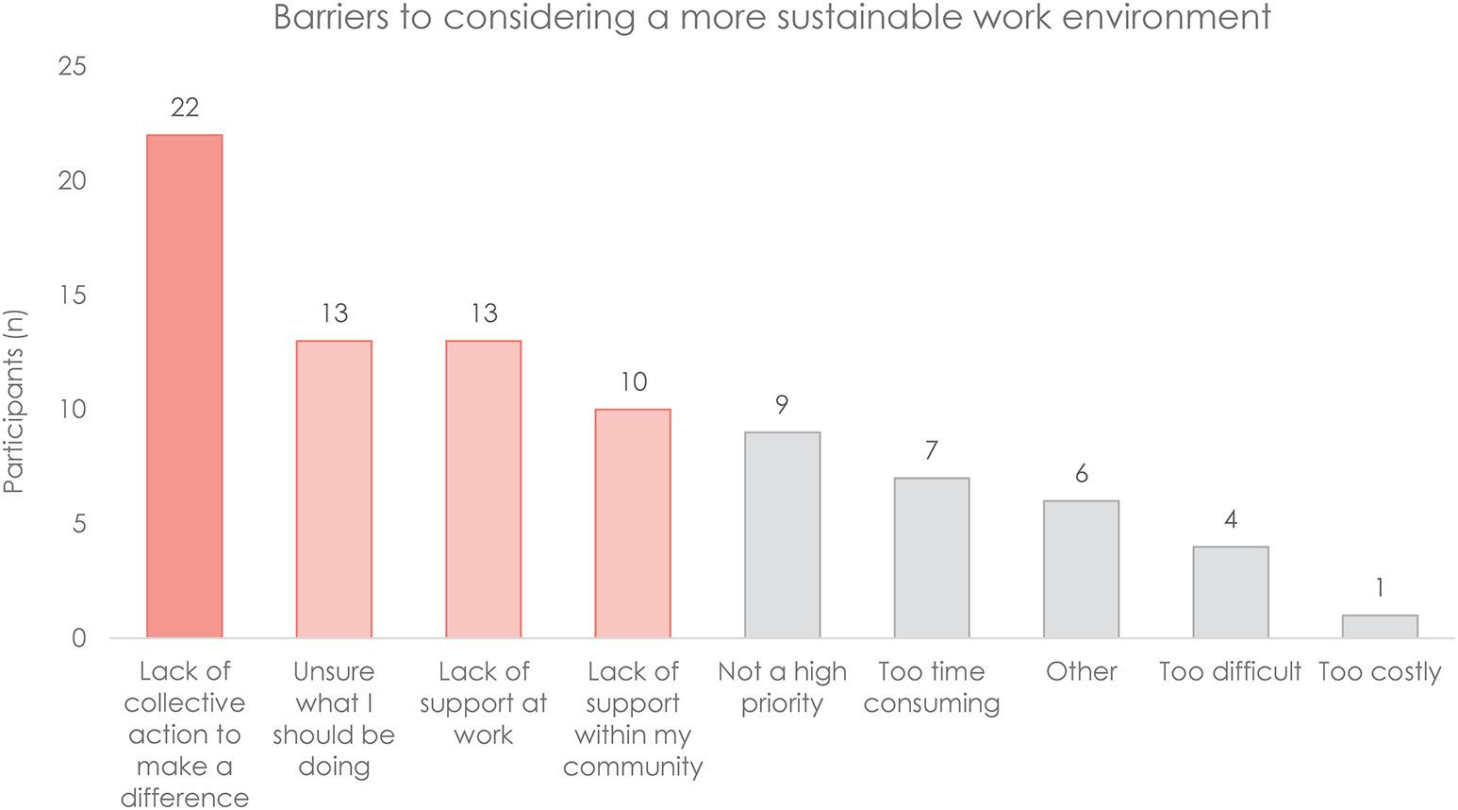
Barriers to considering a more sustainable environment at work. Participants were asked to select barriers that prevented them from adopting sustainable practices at work, participants could select more than one barrier (*n* = 37), *y*-axis represents the absolute number of participants.

Survey participants were asked to identify barriers to creating a more sustainable environment at work (Figure 3). The primary barriers identified were a lack of collective action (59.5%; 22 out of 37), uncertainty about what actions to take (35.1%; 13 out of 37), and lack of support at work (35.1%; 13 out of 37) and within their communities (27%; 10 out of 37). Six participants reported other specific barriers which were: limited time and workload constrain (‘so much to do, and so little time’), general lack of acceptance or resistance to change (‘not generally accepted’), regional or local constraints implying specific geographic challenges, a belief that current efforts within the given framework were already maximized (‘we are doing our best’), and inadequate transportation infrastructure, making it difficult to encourage the use of public transport (‘more widely, the transport network is not good enough to encourage people to use public transport’).

### 3.4 Insights on hospital sustainability

**Figure 4:**
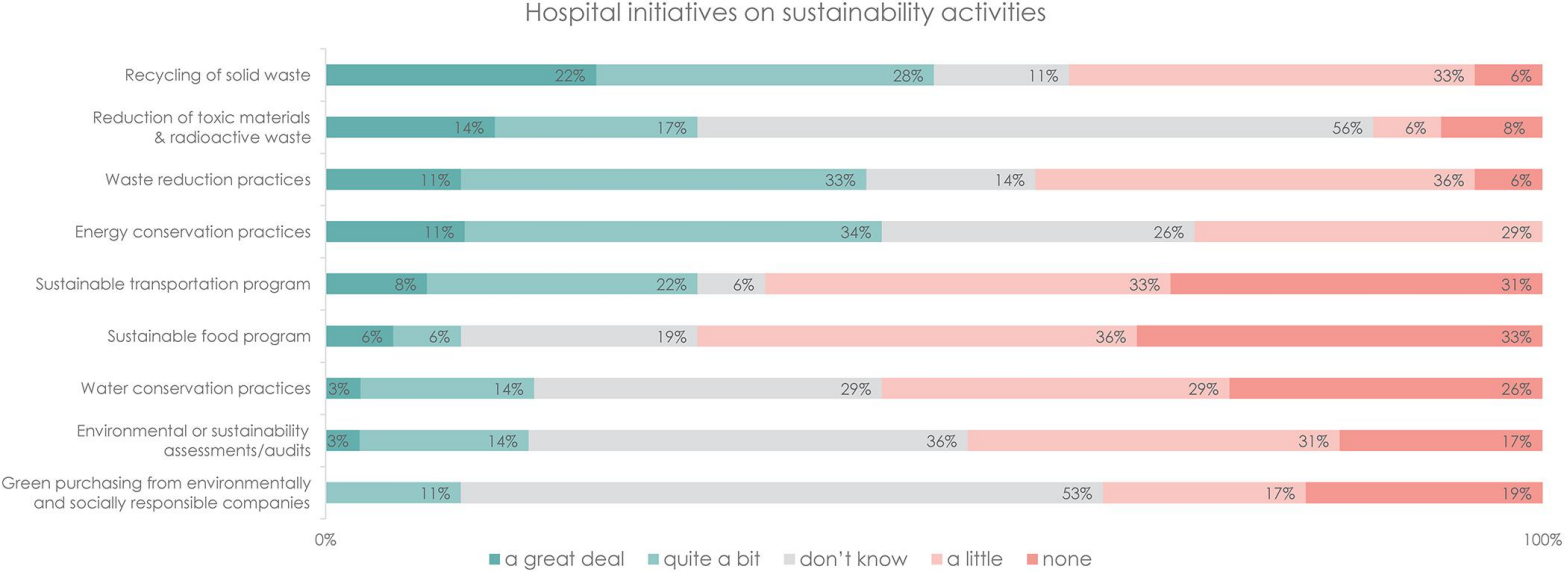
Awareness of sustainable practices currently implemented in the hospital and level of engagement in these practices. Participants were asked to rate their hospital's involvement in the proposed sustainability activities, possible answers were: a great deal, quite a bit, a little, none, and don't know (*n* = 36 except for ‘water conservation practices’ and ‘energy conservation practices’ were *n* = 35), *x*-axis represents the percentage of participants.

Most participants (69.4%; 25 out of 36) were aware of sustainable practices currently implemented in their hospital. When asked about the involvement of their hospital in specific sustainability activities, the responses indicated varying levels of initiation and awareness (Figure 4). Recycling of solid waste emerged as the most widely recognized initiative, with 83.3% (30 out of 36) of participants indicating their hospital had implemented this to at least a minimal extent, including 22.2% (8 out of 36) reporting a ‘great deal’ of engagement. Waste-reduction practices (80.6%; 29 out of 36) and energy-conservation practices (74.3%; 26 out of 35) also demonstrated similarly high levels of implementation, indicating that these areas are central to hospitals’ sustainability efforts. Efforts related to sustainable transportation programs showed moderate implementation, with 63.9% (23 out of 35) of participants reporting at least some level of activity. Water-conservation practices (45.7%; 16 out of 35) and environmental or sustainability assessments/audits (47.2%; 17 out of 36) had comparable levels of implementation. In contrast, green purchasing from environmentally and socially responsible companies showed lower implementation, with 52.8% (19 out of 36) of participants unsure and only 16.7% (6 out of 36) observing at least minimal activity in this area. The reduction of toxic materials and radioactive waste also showed relatively limited implementation, with 55.6% (20 out of 36) of participants indicating they were unaware of any activity. Similarly, sustainable food programs displayed limited adoption, with 33.3% (12 out of 36) reporting no activity and only 11.1% (4 out of 36) indicating moderate or high levels of implementation (‘quite a bit’ or ‘a great deal’).

**Figure 5:**
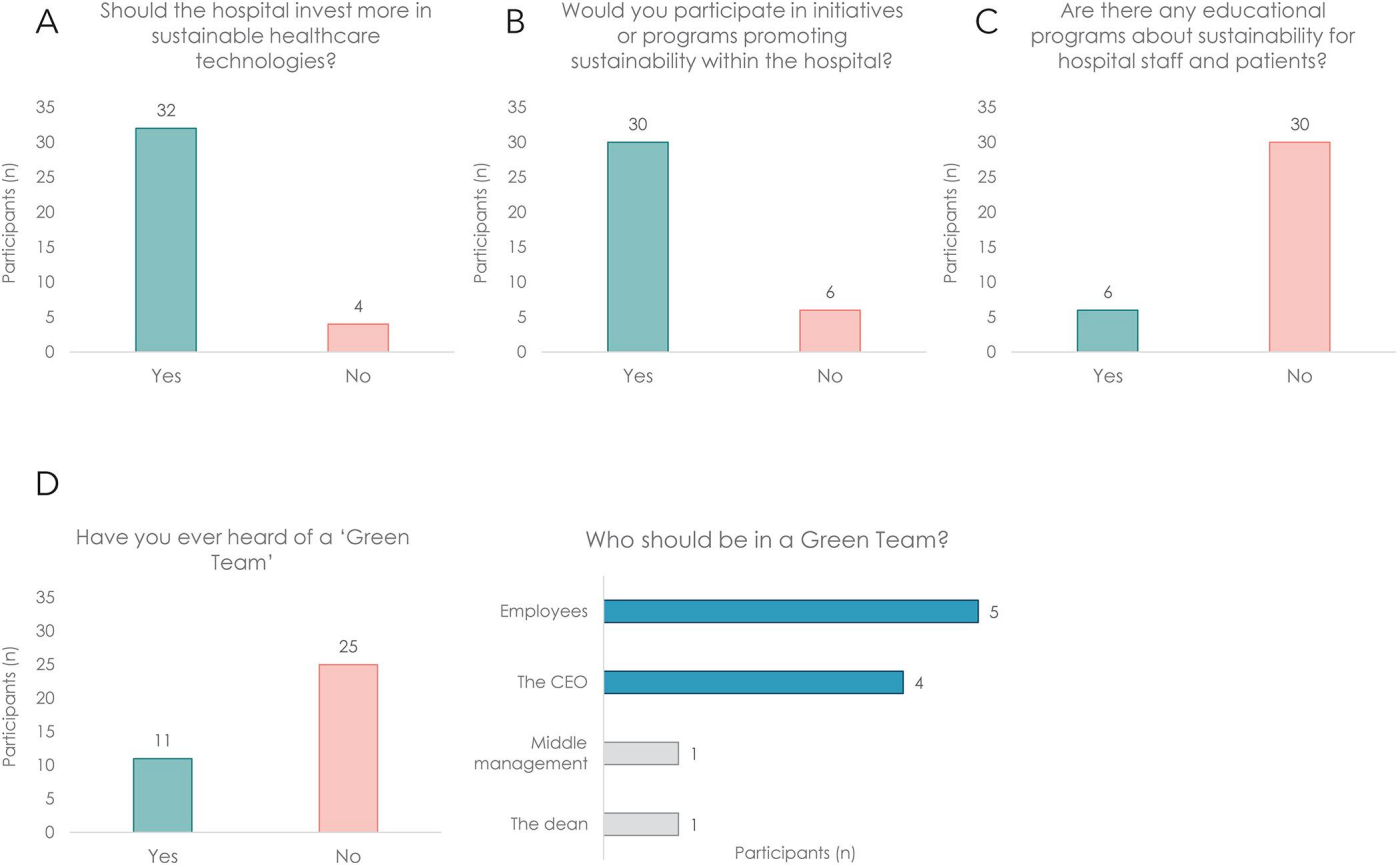
Participants’ responses regarding the hospital's investment on sustainability and willingness to participate in sustainability initiatives at the hospital: **(A)** Do you believe the hospital should invest more in sustainable healthcare technologies? (*n* = 36); **(B)** would you participate in initiatives or programs promoting sustainability within the hospital? (*n* = 36); **(C)** Are there any educational programs about sustainability for hospital staff and patients? (*n* = 36); **(D)** have you ever heard of a ‘green team’ (*n* = 36) and who should be in a green team? (*n* = 11). In panels **(A–C)**, and (**D**, left), the *y*-axis represents the absolute number of participants, in panel (**D**, right) the *x*-axis represents the absolute number of participants.

Almost all the participants believed the hospital should invest more in sustainable healthcare technologies (88.9%; 32 out of 36, Figure 5A), and most of them were willing to participate in initiatives or programs for the promotion of sustainability within the hospital (83.3%; 30 out of 36, Figure 5B). However, there was a notable lack of educational programs on sustainability for hospital staff and patients, with 83.3% (30 out of 36) reporting there were none available (Figure 5C).

Most participants had not heard of a Green Team (69.4%; 25 out of 36, Figure 5D). Eleven participants were familiar with the term, and when asked who should be on a Green Team, the most popular choices were employees (45.5%; 5 out of 11) and the CEO (36.4%; 4 out of 11, Figure 5D). When asked to describe what a Green Team does, 6 participants provided definitions that generally emphasized the role of such teams in promoting and implementing sustainability within the workplace; all reported Green Team responsibilities are given in Table 1.

**Table 1:** Responsibilities of a green team.

What does a Green Team do?
1. Small projects initiated by the employees, but with the support of the management, to make meaningful improvements, e.g., reduction of waste, more efficient use of instrumentation sets, etc.
2. A green team is made up of individuals keen to champion sustainability, working together to generate and implement strategies to promote sustainability in the workplace.
3. Looks for ways to create a more environmentally sustainable work environment.
4. Educates and inculcates values which sensitize the hospital staff towards achieving sustainability goals set by the management
5. Identifies and puts in place practices that support environmental sustainability
6. Evaluates sustainable practices and acts to make improvements at a hospital wide level.

Finally, of the 37 participants, 18 provided suggestions for how their workplaces could promote sustainability. Their suggestions were categorized into 6 key areas. The most frequently mentioned area was waste management, materials, and packaging (61.1%; 11 out of 18), followed by support and infrastructure (38.9%; 7 out of 18), energy and lighting (33.3%; 6 out of 18), and awareness and education (27.8%; 5 out of 18). Identifying key areas for sustainability and remote work and transportation (each 11.1%; 2 out of 18) received fewer mentions.

Participants pointed out the importance of reducing packaging, avoiding single-use materials, and using environmentally friendly alternatives. Suggestions included developing medical consumables from sustainable materials, standardizing recycling systems, and implementing effective waste separation practices to avoid confusion. Several participants emphasized the need for stronger support and infrastructure to facilitate sustainability initiatives. This included increased investment and management support for green teams, sustainable operating theatres and intensive care units, upgrading heating and cooling systems, and making sustainability a part of the organization's mandate. Energy and lighting improvements were also stressed, such as installing more efficient lighting systems, and reducing excessive energy use with measures like automatic lighting and eco-friendly equipment. Awareness and education were seen as essential for fostering sustainable practices. Participants suggested initiatives such as creating educational materials for employees and patients, supporting Green Teams, and producing ‘electronic marketing material to show the benefits of the organization's efforts as a whole – how much was recycled, how much was saved from landfill, what was the reduction in the carbon footprint, how much water was saved, etc. so that employees can better understand and appreciate the value of their efforts’.

## 4 Discussion

Sustainability in healthcare is increasingly becoming a critical priority. This is the first study to investigate the understanding, engagement, and opportunities for sustainability among ORL professionals, particularly within the field of hearing care and cochlear implantation. As healthcare systems strive to reduce their environmental footprints, findings from this survey contribute to a growing body of evidence highlighting the importance of specialty-specific strategies to address sustainability challenges.

The high level of interest in sustainability among survey participants demonstrates a clear acknowledgment of the need for environmentally responsible practices within the field. However, the observed gap in confidence regarding the ability to explain the sustainable development concept suggests that while interest is strong, there is a need for targeted education and training to enhance understanding and practical implementation of sustainability principles. This aligns with observations in surgical and anaesthesia-related fields, where knowledge of sustainability principles does not always translate into actionable practices (8, 22). Training ORL practitioners on sustainability principles through workshops, online modules, or inclusion in clinical curricula can be pivotal. The demonstrated effectiveness of sustainability education in radiography, for example, highlights its potential utility across other disciplines (13). Furthermore, recent initiatives in postgraduate medical education and surgical fellowship training have demonstrated the feasibility and value of incorporating healthcare sustainability into clinical training programs (14, 15). Similar approaches may help equip future ORL professionals with the knowledge and practical skills needed to implement sustainable healthcare practices.

Incorporating local anaesthesia in cochlear implant (CI) surgeries could further enhance sustainability efforts. Local anaesthesia, as opposed to general anaesthesia, can reduce the environmental impact of surgical procedures by minimizing the use of anaesthetic gases, which are known to have significant greenhouse gas effects (23, 24). Total intravenous anaesthesia (TIVA), often considered as a more sustainable alternative to volatile anaesthetics, also presents certain environmental challenges. Agents like propofol, commonly used in TIVA, can be toxic to aquatic life if improperly disposed of (24). Additionally, local anaesthesia can lead to shorter recovery times and reduced postoperative care, thereby decreasing the overall resource use and waste generation associated with CI surgeries (25–27). This approach not only aligns with sustainability goals but also offers potential benefits for patient safety and comfort.

Beyond surgical interventions, post-operative care strategies such as remote CI programming offer further opportunities to advance sustainability in hearing healthcare. The efficacy and safety of remote programming has been demonstrated across all age groups (28), with substantial environmental and economic benefits also reported (29). Remote CI services, whether delivered via portable laptops or hosted remote sites, have been shown to substantially reduce travel-related carbon emissions, with savings of approximately 110–170 kg CO_2_ per appointment. These reductions are accompanied by notable cost savings for the healthcare system, as demonstrated in the province of Ontario, Canada (29). Integrating remote CI rehabilitation into standard care pathways represents a practical, scalable solution that aligns with both environmental goals and patient-centred care.

Building an institutional framework to implement sustainability programs is equally important. Participants’ descriptions of the responsibilities of a Green Team suggest that those familiar with the concept view these teams as collaborative, proactive groups that can identify and implement strategies to promote environmental sustainability while fostering a culture of awareness. The effectiveness of a multidisciplinary Green Team has been demonstrated in a waste-separation pilot study done in a single operating theatre. The Green Team promoted the ‘5Rs’: Reduce, Reuse, Recycle, Rethink, and Research and the study reported a substantial reduction in CO_2_ emissions and cost savings due to decreased incineration of biohazardous waste (30). In ORL care, however, the notable lack of familiarity with Green Teams indicates an overlooked opportunity to organize and promote sustainability initiatives. Establishing multidisciplinary Green Teams in hearing care settings could play an important role in coordinating interventions, raising awareness, and ensuring collective accountability.

Hospitals appear to prioritize initiatives such as recycling, waste reduction, and energy conservation, with these activities being the most consistently implemented across respondents. This is consistent with observations in other healthcare specialties (31, 32). In contrast, initiatives such as green purchasing, toxic-waste reduction, water conservation, and sustainable food programs displayed greater variability and lower levels of engagement. These findings suggest that while hearing care hospitals are progressing in certain key areas, broader and more comprehensive implementation is needed. Moreover, the high levels of uncertainty among participants regarding their hospital's sustainability actions point to a communication gap. Sharing the successes and outcomes of sustainability initiatives could enhance staff engagement, instil a sense of accomplishment, and encourage broader participation. A Green Team could also play a central role in bridging this gap, acting as both educators and champions of hospital-wide sustainability efforts.

The majority of participants made suggestions related to waste management and materials. One potential strategy on this regard is implementing circular economy principles in hospitals. ‘Zero Waste Europe’ is an initiative advocating for the implementation of circular economy principles (33). These principles focus on establishing a waste hierarchy that prioritizes prevention by rethinking and redesigning processes to minimize resource use. When prevention is not feasible, reusing products in their original form or preparing them for reuse is encouraged. Only after these steps have been exhausted should recycling or material and chemical recovery be considered. Practical strategies for ORL clinics and hospitals to adopt circular economy principles include reducing the reliance on single-use items wherever possible and transitioning to sterilizable and reusable alternatives. Similarly, single-use plastics, such as those used for syringes, gloves, or packaging, could be reevaluated to explore opportunities for biodegradable or reusable variants. Overall, integrating circular economy strategies into hospital operations can reduce waste generation, costs, and carbon emissions while improving resource efficiency (34, 35). A shift toward energy-efficient equipment for cleaning and sterilizing reusable items and water conservation measures could further reduce the carbon footprint. However, more research is needed to determine if the energy and water demands of sterilization processes outweigh the environmental costs of disposal in all cases (7). Repurposing materials is another critical component of circular economy thinking. For example, durable medical equipment can be considered for refurbishment programs, where select parts are inspected, refurbished, and redistributed for reuse. Moreover, hospitals could adopt refill and reuse systems for certain consumables, such as disinfectants, gels, or liquid soaps, which are traditionally supplied in disposable plastic containers. Non-clinical materials, such as furniture or administrative tools like binders and file folders, can also be refurbished or repurposed to extend their functional lifespan. Lastly, hospitals can implement robust waste segregation systems to ensure materials are appropriately sorted for recycling or recovery. For instance, separated waste streams for glass, metals, and certain plastics can facilitate more efficient recycling. Dedicated bins for hazardous and non-hazardous medical waste help reduce unnecessary incineration of items that could otherwise be recycled or reused. Initiatives like composting biodegradable waste from hospital cafeterias or collaborating with local food banks to reduce food waste can complement clinical waste-reduction efforts. By integrating these practical measures into daily operations, ORL clinics and hospitals can align with circular economy principles, reducing their environmental footprint while maintaining high standards of care (30). Combining these actions with institutional support and staff training ensures that sustainability efforts are effectively sustained.

At an organizational level, hospitals and clinics can also assess the materials they purchase and prioritize partnership with environmental conscious suppliers. Life Cycle Assessment (LCA) is an essential tool for this purpose. LCA is a systematic methodology for evaluating the environmental impacts associated with all stages of a product, process, or service's life cycle. It helps identify and quantify all inputs (e.g., raw materials, energy) and outputs (e.g., emissions, waste) across the full spectrum of a products life, from raw material extraction to disposal or recycling. LCA is increasingly performed in pharmaceutical and clinical applications (6). Hospitals and clinics, which rely on a wide range of medical devices, supplies, and pharmaceuticals, can use LCA to compare the environmental impacts of products and make evidence-based decisions on which products are more sustainable over their entire life cycle. Through such assessments, hospitals can better align their operational practices with sustainability objectives and move towards more circular and environmentally responsible systems (6).

This study's global scope represents perspectives from diverse healthcare systems across 16 countries, and its comprehensive focus highlights both individual engagement and institutional sustainability actions. A recent bibliometric analysis identified the 10 most research-active countries in in sustainable healthcare research (36). Of these, participants in the present study were drawn from eight of the top ten countries, indicating that the sample includes representation from the majority of highly active regions in sustainable healthcare research. Using Sustainable Development Goal (SDG)-based national rankings as a general indicator of sustainability performance (37), the majority of participants were affiliated with countries classified as having high overall SDG scores. Specifically, approximately two-thirds of participants were based in high-ranking countries, with smaller proportions from medium- and lower-ranking countries. While these indices are not specific to healthcare systems, they provide a broad contextual perspective on the sustainability landscape represented in the sample. However, the study is limited by its reliance on self-reported data, which may be subject to bias, particularly with socially desirable responses. The absence of objective measures, such as hospital audits or regional-policy reviews, limits the ability to fully evaluate the effectiveness of hospital sustainability practices. Moreover, the survey population consisted of members of the HEARRING group, and respondents and authors were not independent groups as explained in section 2.1. Although survey responses were collected before manuscript preparation and results are presented descriptively without inferential analyses, the study is susceptible to participation bias, social desirability bias, and interpretative bias. Findings should therefore be interpreted as reflecting the views of a self-selected group of engaged experts and this should be considered when interpreting the results. Despite these limitations, the findings provide important groundwork for advancing sustainable initiatives in the field of hearing care and promoting sustainable healthcare practices worldwide.

Moving forward, specialized strategies tailored to ORL surgery and cochlear implantation are needed. Investments in sustainable surgical practices, greener operating theatres, reusable materials, and staff engagement must be paired with education and leadership support to accelerate progress. Green Teams could serve as a valuable driver for change in this field, particularly if they are supported with sufficient resources and leadership backing.

National boards and associations can also play a crucial role in driving sustainability initiatives. Organizations like ENT UK (the professional membership body representing ear, nose and throat surgery) have been actively promoting sustainability practices within ORL care. In their recent guidelines, they emphasize the use of reusable instruments and minimizing unnecessary waste in ORL procedures such as wax microsuction (the removal of cerumen obturans) and flexible nasoendoscopy (38–40). Similarly, the British Association for Paediatric Otorhinolaryngology (BAPO) is exploring ways to reduce waste, such as eliminating gowns for tonsillectomies (41). A recent survey in 63 ORL specialists showed that the majority would consider wearing reusable gowns, provided they are properly sterilized between procedures (42). National associations should lead efforts to discuss and collaborate with governments to implement sustainable practices across healthcare systems. In addition, organizations like the HEARRING group can promote sustainability by motivating healthcare professionals to take action and by advocating for changes in healthcare systems to reduce environmental impact and improve resource efficiency. By setting standards and advocating for policy changes, these boards and organizations can significantly influence the adoption of sustainable practices in healthcare.

## 5 Conclusion

While there are promising levels of interest and engagement among ORL professionals, systemic barriers, knowledge gaps, and limited organizational support can hinder progress. Addressing these challenges will require targeted educational programs on zero waste principles, investments in sustainable infrastructure, and leadership-driven initiatives. Green Teams have the potential to drive transformative change, provided they receive adequate resources and institutional backing. By empowering ORL professionals to champion sustainability and circular economy principles within their practice, the hearing care field can contribute to integrating environmental responsibility across healthcare systems. The path forward relies on a collective commitment to adopting sustainable practices, fostering a culture of awareness, and developing innovative solutions to reduce the healthcare sector's environmental impact.

## Data Availability

The raw data supporting the conclusions of this article will be made available by the authors, without undue reservation.
