# Supplementary material for: Towards sustainable healthcare in otorhinolaryngology: assessing knowledge, engagement, and opportunities for change
Source: Front Health Serv. 2026 Jul 17;6:1867484. doi: 10.3389/frhs.2026.1867484 (PMC13423895; doi:10.3389/frhs.2026.1867484)
Supplement: Supplementary file 1 [file Table1.docx]

Supplementary Material

# Supplementary Data

- 1. **Supplementary Table 1.** Workspace actions that relate to sustainability: Participants were asked to reflect on their sustainability-related actions at work. Possible answers were *yes*, *sometimes*, or *no*.

| **As a reflection of your actions at work please indicate whether you:** |
| --- |
| 1. Actively minimize your amount of waste |
| 1. Seek to minimize the amount of energy you use |
| 1. Minimize your travel by motorized transport |
| 1. Try to minimize your impact on the environment |
| 1. Walk, cycle, or use public transport as a frequent mode of travel |
| 1. Minimize water usage |
| 1. Grow your own food |
| 1. Consider ethical/environmental values within the goods and services you buy |
| 1. Are actively involved community projects related to sustainability in your spare time |

- 1. **Supplementary Table 2.** Barriers to considering a more sustainable environment at work: Participants were asked to identify one or more barriers to adopting sustainable practices at work. Participants could also select *Other* to provide additional items that they considered as barriers.

| **What are the barriers to you considering a more sustainable environment at work?** |
| --- |
| 1. Unsure what I should be doing |
| 1. Too difficult |
| 1. Too time consuming |
| 1. Too costly |
| 1. Lack of collective action to make a difference |
| 1. Lack of support within my community |
| 1. Lack of support at work |
| 1. Not a high priority |
| 1. Other (please state) |

- 1. **Supplementary Table 3.** Awareness of sustainable practices currently implemented in the hospital and level of engagement in these practices: Participants were asked if they were aware of any sustainable practices currently implemented in their hospital. Participants were asked to rate any sustainability activities taken by their hospital according to a five-point scale. Possible answers were *0 – don’t know*, *1 – none*, *2 – a little*, *3 – quite a bit*, and *4 – a great deal*. Participants could also select *Other* to provide additional sustainability actions.

| **Has your hospital initiated any of these activities?** |
| --- |
| 1. Energy conservation practices (including lighting, heating, cooling, ventilation, windows, etc.) |
| 1. Waste reduction practices (such as e-communications, double-sided copying, “waste free lunch” program, etc.) |
| 1. Recycling of solid waste (including paper, plastic, metal, e-waste, etc.) |
| 1. Sustainable food program (such as local, organic, and/or fair-trade food) |
| 1. Water conservation practices |
| 1. Sustainable transportation program (including bicycle/pedestrian friendly systems, carpools, bus pass programs, biodiesel projects, etc.) |
| 1. Green purchasing from environmentally and socially responsible companies (products are non-toxic, water and energy conserving, etc.) |
| 1. Reduction of toxic materials and radioactive waste |
| 1. Environmental or sustainability assessments/audits |
| 1. Others (please specify) |

- 1. **Supplementary Plain Language Summary**

**Why was the study done?** Hospitals and clinics significantly impact the environment due to their high energy consumption and resource use. Different medical specialties contribute to this impact in unique ways, making it important to develop sustainability solutions tailored to each field. This study focuses on ORL (otorhinolaryngology) professionals.

**What did the researchers do?** We distributed a questionnaire to ORL professionals to assess their familiarity with sustainability and to understand what practices they and their hospitals currently implement to promote sustainability.

**What did the researchers find?** We received 37 responses from 26 hospitals across 16 countries. Most ORL professionals expressed interest in sustainability and a willingness to participate in hospital initiatives aimed at promoting eco-friendly practices. However, the majority noted a lack of educational programs on sustainability for hospital staff and patients. Awareness of Green Teams (groups promoting eco-friendly practices within organizations) was low, but those familiar with them recognized their potential to drive positive change.

**What do the findings mean?** From our findings, we conclude that improved education opportunities, stronger organizational support, and greater leadership involvement are essential to advancing sustainability in ORL care. Green Teams can play a key role in advancing environmentally friendly practices in hearing care, ultimately contributing to the reduction of healthcare’s global environmental footprint.
